# Recombinant mouse periostin ameliorates coronal sutures fusion in Twist1^+/−^ mice

**DOI:** 10.1186/s12967-018-1454-2

**Published:** 2018-04-17

**Authors:** Shanshan Bai, Dong Li, Liang Xu, Huichuan Duan, Jie Yuan, Min Wei

**Affiliations:** Department of Plastic and Reconstructive Surgery, Shanghai 9th People’s Hospital, Shanghai Jiao Tong University School of Medicine, 639 Zhi Zao Ju Road, Shanghai, 200011 China

**Keywords:** TWIST1, Saethre–Chotzen syndrome, Periostin, Wnt/β-catenin pathway

## Abstract

**Background:**

Saethre–Chotzen syndrome is an autosomal dominantly inherited disorder caused by mutations in the twist family basic helix-loop-helix transcription factor 1 (TWIST1) gene. Surgical procedures are frequently required to reduce morphological and functional defects in patients with Saethre–Chotzen syndrome. Therefore, the development of noninvasive procedures to treat Saethre–Chotzen syndrome is critical. We identified that periostin, which is an extracellular matrix protein that plays an important role in both bone and connective tissues, is downregulated in craniosynostosis patients.

**Methods:**

We aimed to verify the effects of different concentrations (0, 50, 100, and 200 μg/l) of recombinant mouse periostin in Twist1^+/−^ mice (a mouse model of Saethre–Chotzen syndrome) coronal suture cells in vitro and in vivo. Cell proliferation, migration, and osteogenic differentiation were observed and detected. Twist1^+/−^ mice were also injected with recombinant mouse periostin to verify the treatment effects.

**Results:**

Cell Counting Kit-8 results showed that recombinant mouse periostin inhibited the proliferation of suture-derived cells in a time- and concentration-dependent manner. Cell migration was also suppressed when treated with recombinant mouse periostin. Real-time quantitative PCR and Western blotting results suggested that messenger ribonucleic acid and protein expression of alkaline phosphatase, bone sialoprotein, collagen type I, and osteocalcin were all downregulated after treatment with recombinant mouse periostin. However, the expression of Wnt-3a, Wnt-1, and β-catenin were upregulated. The in vivo results demonstrated that periostin-treated Twist1^+/−^ mice showed patent coronal sutures in comparison with non-treated Twist1^+/−^ mice which have coronal craniosynostosis.

**Conclusion:**

Our results suggest that recombinant mouse periostin can inhibit coronal suture cell proliferation and migration and suppress osteogenic differentiation of suture-derived cells via Wnt canonical signaling, as well as ameliorate coronal suture fusion in Twist1^+/−^ mice.

**Electronic supplementary material:**

The online version of this article (10.1186/s12967-018-1454-2) contains supplementary material, which is available to authorized users.

## Background

Craniosynostosis, the premature fusion of the cranial sutures, is a significant medical problem that occurs in both syndromic and nonsyndromic cases [1]. The incidence of craniosynostosis is 1 in 2000–3000 live births [2]. Saethre–Chotzen syndrome (SCS; OMIM 101400), also known as acrocephalosyndactyly III (ACS III), is one of the most common syndromic forms of craniosynostosis, which is an autosomal dominant disorder characterized by coronal synostosis, facial asymmetry, ptosis, and limb abnormalities. The symptoms of SCS vary widely, even among affected patients in a single family [3]. It has been demonstrated that mutations in the twist family basic helix-loop-helix (bHLH) transcription factor 1 (TWIST1) gene on chromosome 7 are associated with SCS. The majority of the mutations that have been described result in loss-of-function, leading to functional haplo-insufficiency of TWIST1 [4, 5]. The TWIST1 protein is expressed in the osteo-progenitor cells within the coronal and sagittal sutures and it is thought to be involved in osteoblast proliferation and differentiation [6]. It has been demonstrated that the stem cell population is diminished in the sutures of Twist1^+/−^ mice with craniosynostosis, which suggests that there is a threshold number of suture stem cells required for maintaining suture patency [7].

SCS patients usually have early fusion in coronal sutures. It may lead to brachycephaly and plagiocephaly, late closure of the fontanelles, and raised intracranial pressure. If not corrected, craniosynostosis can result in blindness, deafness, developmental delays, and, in severe cases, death [8]. At present, surgical procedures, such as suturectomy and frontal-supraorbital advancement, have been successful in preventing these abnormalities and symptoms, but rapid resynostosis can increase cranial pressure and further place restrictions on the growing brain and cranial base [9]. Attempts at developing adjuvant treatments and understanding the biological mechanisms that cause craniosynostosis would address a significant need.

We have screened out a series of genes related to skull development and craniosynostosis, noting in particular that periostin was downregulated (superior to 5.8-folds) in craniosynostosis patients, a finding that should acquire much attention. Periostin, also termed osteoblast-specific factor 2, is a matricellular protein with high levels of expression in the periosteum, the layer of connective tissue surrounding bone, and it is responsible for the increase in bone diameter, which is related to bone strength [10]. Periostin is also a novel marker for intramembranous ossification and is expressed in the mesenchymal cells of mouse skulls. Periostin has been observed to play a role in the development of craniosynostosis, as it is expressed in the domain of Twist1, forming both homodimers (T/T) and heterodimers with E2A E proteins (T/E) in the cranial sutures of Twist1^+/−^ mice, and is altered by changes in Twist1 expression, which means there must be interplay between periostin and Twist1 in the cranial suture morphogenesis of Twist1^+/−^ mice [11]. Therefore, we speculated that periostin may be involved in the development of cranial sutures in Twist1^+/−^ mice and it could be a target for an adjuvant therapy to improve craniosynostosis.

To address these unresolved challenges and verify our assumptions, the overall goals of this study were to use recombinant mouse periostin to treat coronal craniosynostosis in a Twist1^+/−^ mice model of SCS. In this study, a murine model was used because fusion of the coronal suture occurs during the first 10 days of age, whereas the remaining sutures stay open. We cultivated suture-derived cells with various concentrations of recombinant mouse periostin to investigate the influence on cell proliferation, migration, and osteogenic differentiation. Meanwhile, Twist1^+/−^ mice were injected daily subcutaneously with recombinant mouse periostin starting at birth (1.0 mg/kg) for 3 weeks to confirm the effect in vivo.

## Methods

### Twist1^+/−^ mice

The SCS Twist1^+/−^ mouse model subjects were provided by Seattle Children’s Hospital (Seattle, WA, USA). Twist1^+/−^ mice were mated with wild-type (Twist1^+/+^) mice to generate the progeny used in this study. Genotyping of tail DNA was used to distinguish mutant from wild-type progeny by polymerase chain reaction (PCR) analysis. The primer sequence of TWSIT1 can be found in Table 1. The mice were killed by cervical dislocation. Twist1^+/−^ mice demonstrated significant craniofacial anomalies as evidenced by micro-computed tomography (CT) studies (Additional file 1: Figure S1). Postnatal 1 day Twist1^+/−^ mice and littermate controls or WT mice were used for phenotypic analyses. The mice were housed in the Shanghai Ninth People’s Hospital’s animal facility (Shanghai, China) and all of the protocols were approved by the Institutional Animal Care and Use Committee of Shanghai Ninth People’s Hospital.

**Table 1:** Final numbers of samples used for each experimental condition

	0 μg/L	50 μg/L	100 μg/L	200 μg/L
Cell proliferation	20	20	20	20
Cell migration	20	20	20	20
Alizarin red staining	6	6	6	6
ALP activity	6	6	6	6
Immunofluorescent staining	6	6	6	6
RT-qPCR analysis	6	6	6	6
Western blotting	6	6	6	6

*RT-qPCR* real-time quantitative PCR

### Isolation and culturing of suture-derived cells

Coronal suture-derived cells were harvested from Twist1^+/−^ mice at postnatal day 1. All the mice were male. Coronal sutures with 500-μm bony margins were dissected with great care to remove all of the dural and pericranial tissue under an anatomical microscope (Olympus, Tokyo, Japan) [12]. Suture explants were cultured in α-minimum essential medium supplemented with 10% fetal bovine serum (Gibco, Gaithersburg, MD, USA), 100 U/ml penicillin (Gibco, Gaithersburg, MD, USA), and 100 μg/ml streptomycin (Gibco, Gaithersburg, MD, USA). The cells were cultured at 37 °C in a 5% CO_2_ humidified incubator and all of the experiments were carried out at a plating density of 50,000 cells/cm^2^. Only suture-derived cells of passage 1 were used.

The cultured suture-derived cells were treated with the following different concentrations of recombinant mouse periostin: 0, 50, 100, and 200 μg/l. The final numbers of samples used for each experiment are presented in Table 1.

All of the cell osteogenic differentiation and matrix mineralization processes were initiated 24 h after plating by replacing the medium with fresh medium supplemented with 50 μg/ml of ascorbic acid (Sigma-Aldrich, St. Louis, MO, USA) and 10 mM β-glycerophosphate (Sigma-Aldrich, St. Louis, MO, USA). The medium was changed once every 3 days and the cells were cultured for 21 days. Upon termination of the culture, cells were only used for alizarin red staining, immunofluorescent staining, and ribonucleic acid (RNA) and protein preparation.

### Cell proliferation assay

Cell proliferation was detected using Cell Counting Kit-8 (CCK-8; Beyotime, Shanghai, China). Suture-derived cells (2000 cells per well) were plated in 96-well plates. Wells containing the standard medium without cells were used as the blanks. The plates were incubated for 1, 3, 5, and 7 days. Then, 20 μl CCK-8 dye solution was added and incubated for 4 h at 37 °C. After 4 h of incubation, optical density values were measured using a micro-plate reader (Thermo Fisher Scientific, Waltham, MA, USA). All of the assays were performed in quintuplicate and repeated using three cell samples.

### Cell migration assay

A monolayer scratch assay was used to compare the migratory ability of suture-derived cells treated with different concentrations of recombinant mouse periostin. Cells were cultured on 6-well plates; upon reaching 90% confluence in the 6-well plates, the cell monolayer was scratched with a sterile 200-μl pipette tip. The cultures were washed with phosphate buffer saline (PBS) to remove detached cells and various concentrations of recombinant mouse periostin were added. Photographs were taken immediately after scratching and again 24 h later. The photographed areas were quantified with computer-assisted image analysis using Image-Pro^®^ Plus 6.0 software (Media Cybernetics, Rockville, MD, USA). The results are presented as a percentage of the area of the scratch filled by the suture-derived cells. The data were acquired from five randomly selected high-power fields.

A transwell system (8-μm pore size; Gibco, Gaithersburg, MD, USA) was used for the assay. Briefly, the cells were harvested and seeded in the upper compartment of the chamber in 24-well plates using a total of 10,000 cells in 0.1 ml of serum-free Dulbecco’s Modified Eagle’s medium with recombinant mouse periostin (0, 50, 100, and 200 μg/l), while the bottom well was filled with 0.6 ml of Dulbecco’s Modified Eagle’s medium plus 10% fetal bovine serum. The cells were then incubated for 24 h. Afterwards, a cotton swab was used to remove the non-migrated cells on the upper surface of the membrane and the migrated cells on the bottom surface of the filter membrane were subsequently fixed in 4% paraformaldehyde and stained with 4′,6-diamidino-2-phenylindole (DAPI). The number of the migrated cells was obtained by counting the stained cells in five randomly selected fields under a microscope (Olympus, Tokyo, Japan).

### Alizarin red staining

After 21 days of culture in osteogenic media, the cells were fixed with methanol for 20 min and stained with 10% alizarin red solution. Mineralized nodules were then identified as red spots on the culture dish.

### Immunofluorescent staining

Suture-derived cells were first cultured in cover slides for 1 day and then fixed with 4% paraformaldehyde in PBS for 15 min at room temperature. After being washed in PBS, the cells were treated with 0.5% Triton X-100 (Bio-Rad, Hercules, CA, USA) for 15 min at room temperature and incubated with normal goat serum for 60 min at 37 °C. Samples were subsequently incubated in rabbit anti-mouse collagen type I (COL-1) antibody (1:50; Santa Cruz Biotechnology, Dallas, TX, USA) at 4 °C overnight. The cells were washed three times with PBS and incubated with secondary antibody (goat anti-rabbit; Invitrogen, Carlsbad, CA, USA) for 1 h at room temperature. After extensive washing in PBS, the cells were mounted with DAPI (Invitrogen, Carlsbad, CA, USA). Micrographs were taken with a Nikon E600 microscope (Nikon, Tokyo, Japan). Analysis was performed using ImageJ software (National Institutes of Health, Bethesda, MD, USA) to evaluate the differences between the 4 groups.

### Alkaline phosphatase activity assay

The activity of intracellular ALP was measured with an ALP activity assay kit (Sigma, St. Louis, MO, USA) with enzyme labelling method, according to the manufacturer’s instructions. The measurements were compared to those of an alkaline phosphatase standard and normalized using the total protein levels obtained with the BCA protein assay kit (Biomiga, Santiago,USA).

### RNA isolation and real-time quantitative PCR

Total RNA was extracted from mice suture tissues or cultured cells using the TRIzol™ Reagent (Thermo Fisher Scientific, Waltham, MA, USA) and complementary DNA was synthesized from 1 μg total RNA per sample using avian myeloblastosis virus reverse transcriptase (Takara Bio Inc., Kusatsu, Japan). The reaction was made in a final volume of 20 μ with 4 μl of magnesium chloride (MgCl_2_), 4 μl of 5× buffer, 2 μl of deoxyribonucleotide triphosphate, 0.5 μl of RNase inhibitor, 1 μl of oligo-(dT), and 1 μl of avian myeloblastosis virus reverse transcriptase, using double-distilled water (ddH_2_O) to meet the final volume. The mixture was incubated at 30 °C for 10 min, 45 °C for 60 min, 98 °C for 5 min, and 5 °C for 5 min.

The designed primers for real-time qPCR analyses are listed in Table 2. PCR was performed with incubation at 95 °C for 3 min, followed by 30 cycles (30 s at 95 °C, 30 s at 60 °C, and 45 s at 72 °C) and subsequent termination by a 5-min extension at 72 °C and storage at 4 °C until analysis. PCR products were separated using 0.75% agarose gels in Tris–acetate-ethylenediaminetetraacetic acid buffer and subsequently photographed under ultraviolet light. β-actin gene was used as an internal control. Experiments were repeated in three tissue samples.

**Table 2:** Primer sequence

	Primer sequence
Mouse TWIST1	
Forward	5′ GGACAAGCTGAGCAAGATTCA 3′
Backward	3′ CGGAGAAGGCGTAG-CTGAG 5′
Mouse AKP	
Forward	5′ CGGATAACGAGATGCCACCAGAG 3′
Backward	3′ CAGTTCAGTGCGGTTCCAGACAT 5′
Mouse BSP	
Forward	5′ AGCGACGAGGAAGAGGAAGAGG 3′
Backward	3′ TTGGTGCTGGTGCCGTTGAC 5′
Mouse COL-1	
Forward	5′ CAGGGCGACAGAGGCATAAAGG 3′
Backward	3′ GGACCAACAGGACCAGCATCAC 5′
Mouse OCN	
Forward	5′ CCCTCACACTCCTCGCCCTATT 3′
Backward	3′ TTCACTACCTCGCTGCCCTCCT 5′
Mouse RUNX-2	
Forward	5′ TGCTGCAGTGATGTGGTTTTCT 3′
Backward	3′ CCCCTGTTGTGTTGTTTGGTAA 5′
Mouse Wnt 7b	
Forward	5′ CTTCACCTATGCCATCACGG 3′
Backward	3′ TGGTTGTAGTAGCCTTGCTTCT 5′
Mouse Wnt 1	
Forward	5′ GGTTTCTACTACGTTGCTACTGG 3′
Backward	3′ GGAATCCGTCAACAGGTTCGT 5′
Mouse Wnt 3a	
Forward	5′ CTCCTCTCGGATACCTCTTAGTG 3′
Backward	3′ CCAAGGACCACCAGATCGG 5′
Mouse β-catenin	
Forward	5′ AAGTTCTTGGCTATTACGACA 3′
Backward	3′ ACAGCACCTTCAGCACTCT5′
Mouse β-actin	
Forward	5′ GCGGGAAATCGTGCGTGACA 3′
Backward	3′ GGAAGGAAGGCTGGAAGAGTGC 5′

*TWIST1* twist family basic helix-loop-helix transcription factor 1, *ALP* alkaline phosphatase, *BSP* bone sialoprotein, *COL-1* collagen type I, *OCN* osteocalcin, *RUNX-2* runt-related transcription factor 2

For qPCR, complementary DNA was amplified using a Power SYBR^®^ Green PCR Master Mix (2×) (Applied Biosystems, Foster City, CA, USA) in a real-time thermal cycler (Stratagene Mx3000PTM QPCR System, Palo Alto, CA, USA). qPCR was conducted in triplicate for each sample and experiments were repeated in three tissue samples. Gene expression was normalized to β-actin expression using the 2^−△△C(t)^ method [13].

### Western blotting

Using equal amounts of protein extracts in a lysis buffer containing 100 mM Tris hydrochloric acid pH 9.0, 200 mM potassium chloride, 25 mM egtazic acid, 36 mM MgCl_2_ 2% deoxycholic acid, and 10% dithiothreitol v/v. AS for β-catenin, the extraction and isolation of nuclear and cytoplasmic protein was performed according to protocols of the Nuclear and Cytoplasmic Protein Extraction Kit (Beyotime, Jiangsu, China). First, cells were centrifuged for 5 min at 1200 rpm at 4 °C and the pellet was dissolved with cytoplasmic protein extraction agent A supplemented with phenylmethane sulfonyl fluoride (PMSF). After vortexing for 5 s, the tubes were incubated for 10–15 min on ice to promote lysis. Next, the cytoplasmic protein extraction agent B was added, vortexing was performed for 5 s, and each sample was incubated on ice for 5 s. Then, the samples were centrifuged for 5 min at 14,000*g* at 4 °C and the supernatant, consisting of the cytosolic fraction, was immediately frozen for further analysis. The pellet was re-suspended in nuclear protein extraction agent supplemented with PMSF. Lastly, after vortexing the tubes between 15 and 20 times for 30 min and centrifuging for 10 min at 14,000*g*, the supernatants containing the nuclear extracts were obtained.

The prepared protein was subjected to sodium dodecyl sulfate polyacrylamide gel electrophoresis and subsequently transferred onto polyvinylidene difluoride membranes. The membranes were incubated with primary antibodies, followed by incubation with appropriate horseradish peroxidase-conjugated secondary antibodies (Jackson ImmunoResearch Laboratories, Inc., West Grove, PA, USA). The protein bands were eventually visualized using an enhanced chemiluminescence detection kit (Amersham, Little Chalfont, UK). The primary antibodies against alkaline phosphatase (ALP), bone sialoprotein (BSP), COL-1, osteocalcin (OCN), runt-related transcription factor 2 (RUNX-2), Wnt-7b, Wnt-3a, Wnt-1, and β-catenin were all purchased from Invitrogen (Carlsbad, CA, USA). The band intensity were quantified using Image J software (version 2.1.4.7, NIH, USA).

### In vivo treatment

All of the care and procedures for the study mice were conducted in accordance with the Institutional Animal Care and Use Committee of Shanghai Ninth People’s Hospital. The age of the mice were postnatal 1 day, all the mice were male. The coronal sutures of Twist1^+/−^ mice (n = 10) were injected daily subcutaneously with 200 μl of collagen mixed with 1.0 mg/kg of periostin. The coronal sutures of Twist1^+/−^ mice (n = 10) were injected daily subcutaneously with 200 ul of collagen. An auto-micro injector was used (Hamilton, Bonaduz, Switzerland) to delivery the periostin, and one second injection of 5 μl. The coronal sutures of wild-type mice (n = 10) were injected daily subcutaneously with 200 μl of collagen with periostin. All of the animals received subcutaneous injections for 3 weeks.

### Micro-computed tomography and coronal suture bone density analyses

All of the animals were killed by an overdose of pentobarbital at 3 weeks to obtain the skulls. After retrieval, the samples were fixed in 10% formalin for at least 48 h. Whole skulls were scanned using micro-CT (Scanco Medical, Bassersdorf, Switzerland). The scanning parameters were set at 70 kV and 114 μA with an exposure time of 400 ms and a resolution of 20 μm. The coronal sutures of mice was chosen as region of interest (ROI) and BV/TV (Bone Volume Fraction) was calculated using the micro-CT system software package. Three-dimensional images of the specimens were reconstructed by Mimics 10.0 software (Materialise NV, Leuven, Belgium).

### Histology

After imaging with micro-CT, the skulls were isolated and fixed in 4% paraformaldehyde in PBS for 24 h at 4 °C and they were then dehydrated in a graded series of ethanol, embedded in paraffin, and sectioned at 7-mm intervals. After the sections were dewaxed and rehydrated, they were stained with hematoxylin and eosin (Wako Pure Chemical Industries, Ltd., Osaka, Japan).

### Statistical analysis

After confirming the normal distribution of the data, homogeneity of variance was tested by Levene’s test. All of the data are presented as means ± standard deviations (SD) and the statistical analyses were completed using the statistical software Statistical Package for the Social Sciences (SPSS version 19.0; IBM Corp., Armonk, NY, USA). A two-tailed Student’s *t* test was used to compare two groups and an analysis of variance test was used to compare three or more groups. Differences with a p value of < 0.05 were considered to be statistically significant. Graphs were created using GraphPad Prism v6.00 for Windows (GraphPad Software, La Jolla, CA, USA).

## Results

### Cell proliferation

**Fig. 1:**
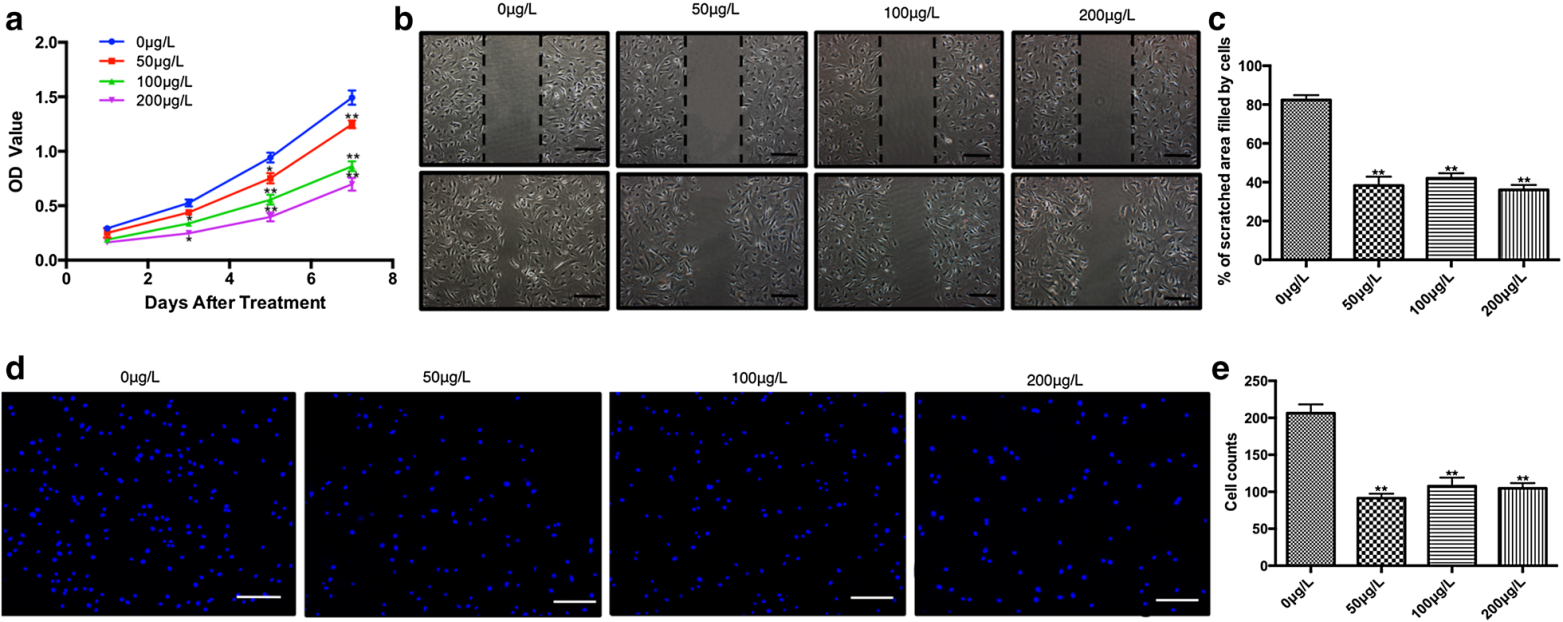
Treatment with periostin inhibits  suture-derived cell proliferation and migration. **a** The cell viabilities of suture-derived cells treated with periostin in different concentrations were measured with a CCK-8 assay at days 1, 3, 5, and 7. **b**, **c** The suture-derived cells were grown to a confluent monolayer, wounded by manual scratching with a sterile pipette tip, and subjected to further incubation with periostin. The scratched areas filled by migrated suture-derived cells were observed at 24 h post-scratching and quantified using Image-Pro Plus software. Original magnifications: ×40. Bar: 100 μm. **d**, **e** As determined by transwell assay, the migratory suture-derived cells were visualized by imaging the nuclei labeled with DAPI. The number of migrated cells was counted in five randomly selected fields. Original magnifications: ×40. Bar: 100 μm. *P < 0.05, **P < 0.01 compared to 0 μg/L group

The aberrant proliferation of suture-derived cells that has been shown to contribute greatly to craniosynostosis [14], we then investigated the ability of periostin to modulate the proliferation of the suture-derived cells. As determined by CCK-8, a colorimetric assay used to measure cell viability and cytotoxicity, periostin significantly inhibited the proliferation of suture-derived cells in concentration- and time-dependent manners as compared with the non-treated group (Fig. 1a). After treatment with periostin for 3 days, the optical density (OD) values in the 100 μg/l group and the 200 μg/l group showed a significant difference when compared with the value in the 0 μg/l group (p < 0.05). There were even great differences in the 100 μg/l group and the 200 μg/l group following treatment with periostin for 5 and 7 days (p < 0.01). The inhibition effect was most significant when the concentration was 200 μg/l; however, the differences between the 100 μg/l group and 200 μg/l group were not significant.

### Cell migration

Suture-derived cells in the 0 μg/l group efficiently migrated into the scratched area (Fig. 1b). In contrast, the migration capacity of suture-derived cells was evidently abrogated in the other three groups (p < 0.01, Fig. 1c), yielding values of only 35.65 ± 3.33% (50 μg/l), 38.09 ± 6.19% (100 μg/l), and 32.74 ± 2.98% (200 μg/l). However, the scratched area of 0 μg/l group was filled after treatment with recombinant mouse periostin. These data were further validated by a transwell assay. As observed in Fig. 1d, recombinant mouse periostin may strikingly reduce the number of cells that migrates across the filter membrane to the bottom surface. Compared with the 0 μg/l group (average 207 ± 7 cells, n = 3), periostin can lead to a noticeable reduction in the number of migrated cells—specifically, approximately 50% of that in the other three groups (p < 0.01, Fig. 1e).

### Periostin suppressed the osteogenic differentiation of suture-derived cells

**Fig. 2:**
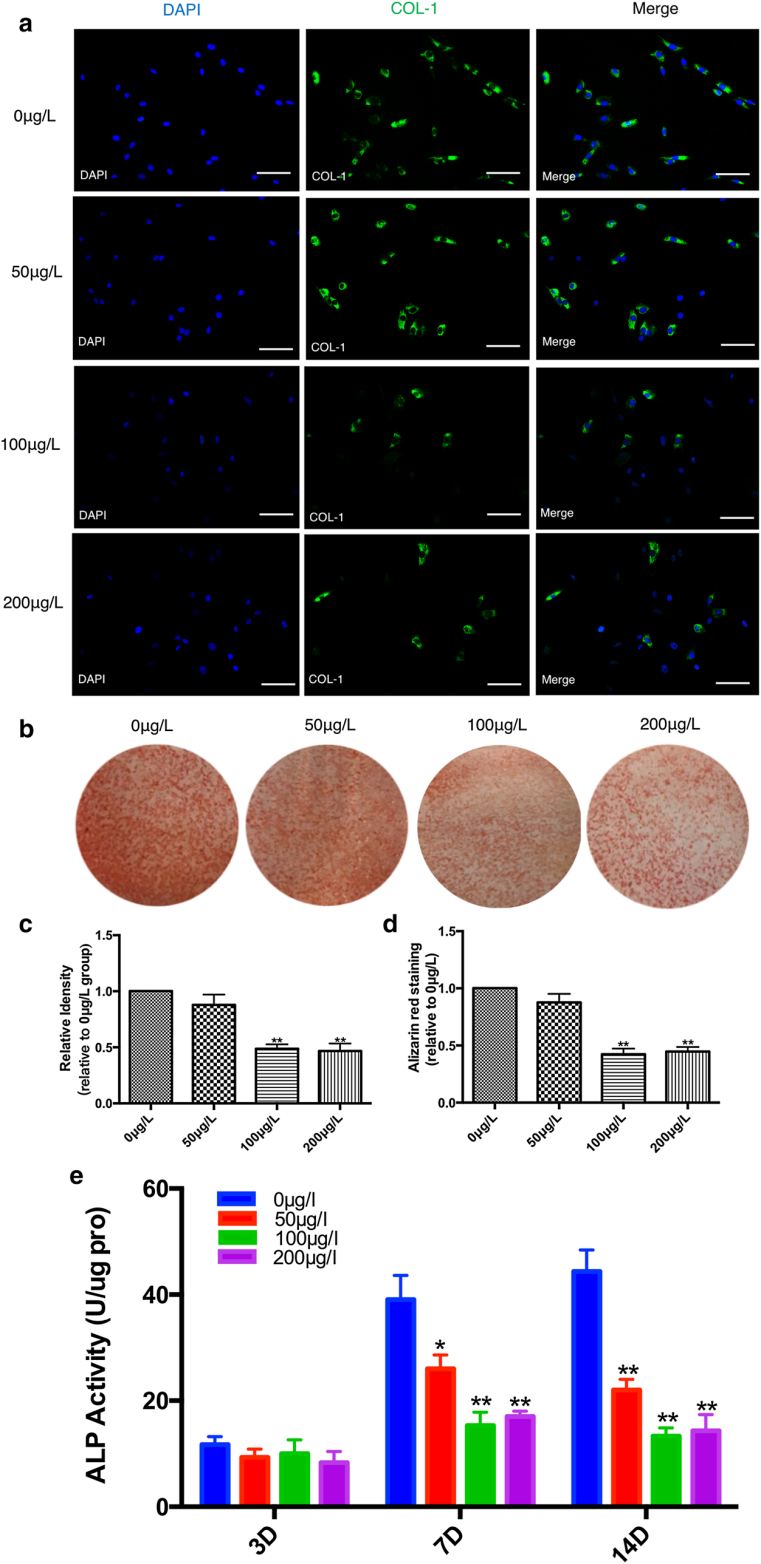
The effect of periostin on suture-derived cells’ mineralization. **a** After treatment with periostin for 21 days, immunofluorescence shows COL-1 was decreased in the suture-derived cells. Original magnifications: ×200. Bar: 50 μm. **b** Quantification of immunofluorescence with IPP software. **c** After treatment with periostin, alizarin red staining showed that the mineralized nodule decreased. **d** Quantification of alizarin red staining of suture-derived cells treated with periostin. **e** ALP activity of suture-derived cells treated with periostin. *P < 0.05, **P < 0.01 compared to 0 μg/L group

To determine how recombinant mouse periostin affects the osteogenic differentiation of suture-derived cells, we performed immunofluorescent staining to measure the expression of COL-1. Our results suggest that the expression of COL-1 decreased significantly in the 100 μg/l group and the 200 μg/l group (p < 0.01, Fig. 2b). However, almost all of the cells revealed green positive signal in the 0 μg/l group (Fig. 2a). These results demonstrated that periostin may suppress the osteogenic differentiation of suture-derived cells. To confirm our results, we also performed alizarin red staining; the subsequent findings showed that periostin decreased the mineralization nodules of these cells after 21 days of incubation (Fig. 2c). Quantification of alizarin red staining of suture-derived cells in the 100 μg/l group and 200 μg/l group showed a significant decline in comparison with in the 0 μg/l group (p < 0.01, Fig. 2d).

ALP activity was also performed to observe the effects of recombinant periostin on the suture-derived cells. As shown in Fig. 2e, when the cells were treated with periostin and cultured for 3 days, there is no significant difference between 0 μg/l group, 50 μg/l group, 100 μg/l group, 200 μg/l group. After 7 days of culture, AlP activity was inhibited in periostin treated group compared to 0 μg/l group (p < 0.05). When culturing for 14 days, the ALP in 50 μg/l group (21.83 ± 3.42) U/μg pro, 100 μg/l group (11.89 ± 2.74) U/μg pro, 200 μg/l group (13.91 ± 6.95) U/μg pro, which are down-regulated obviously compared to 0 μg/l group (46.72 ± 8.51) U/μg pro.

**Fig. 3:**
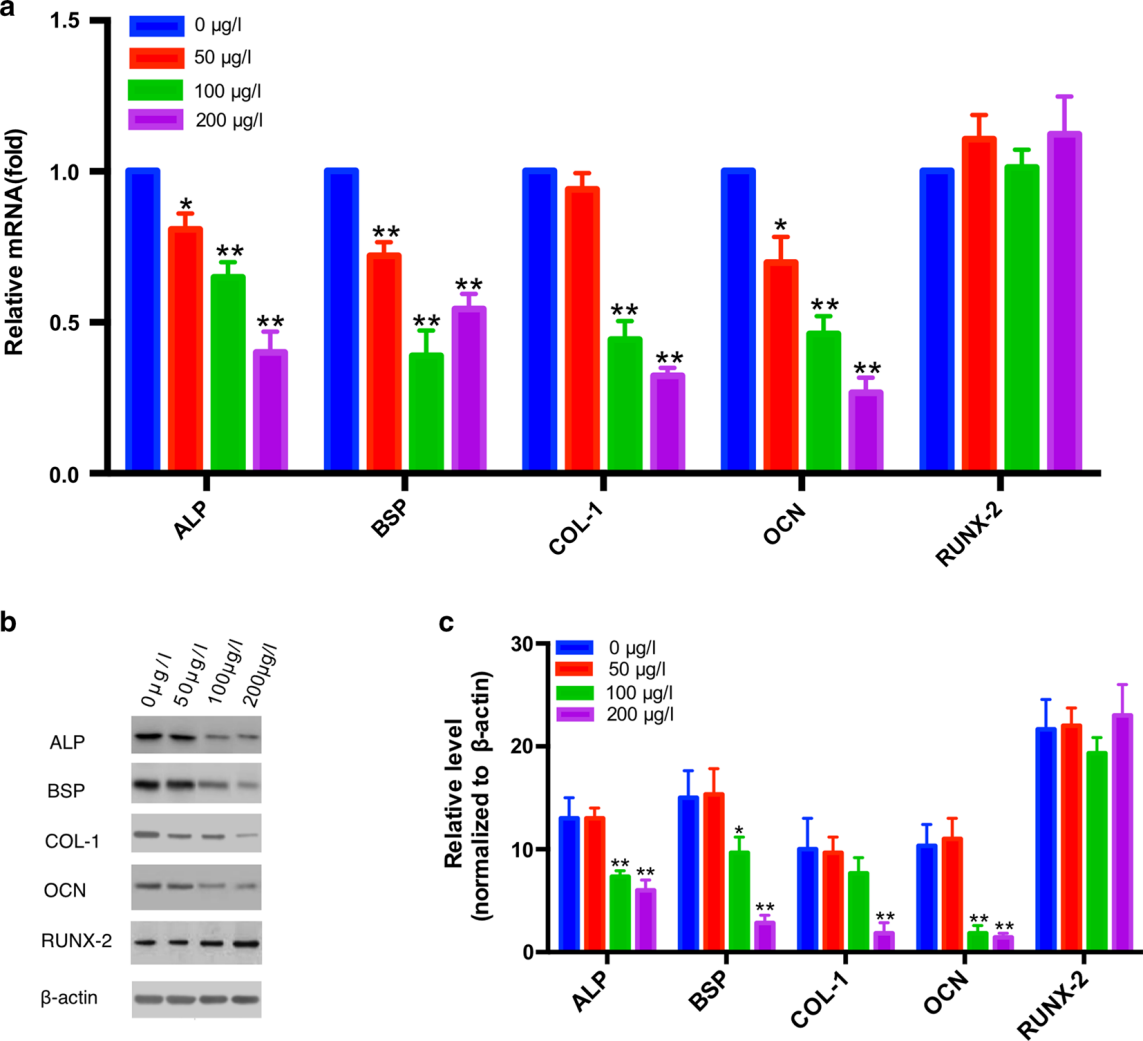
Periostin suppressed the osteogenic differentiation of suture-derived cell. **a** The mRNA expression of ALP, BSP, COL-1, OCN, and RUNX-2 of suture-derived cells after treatment with different concentrations of recombinant mouse periostin. **b** Western blotting analysis of osteogenic differentiation markers. **c** Quantification of Western blotting results. *P < 0.05, **P < 0.01 compared to 0 μg/L group

After 21 days of culture, RNA from suture-derived cells was used to measure the levels of the expression of genes related to osteogenic differentiation using qRT-PCR. As shown in Fig. 3a, the expression levels of ALP, BSP, COL-1, and OCN in the 50 μg/l group, 100 μg/l group, and 200 μg/l group were all lower versus those in the 0 μg/l group (p < 0.05). Additionally, Western blotting analyses were consistent with RT-PCR (Fig. 3b, c), which indicated that recombinant mouse periostin inhibited both transcriptional and translational levels of osteogenic differentiation markers of suture-derived cells. However, the expression of RUNX-2 varied minimally (Fig. 3a–c).

### Periostin suppressed the osteogenic differentiation of suture-derived cells via activation of Wnt/β-catenin signaling

**Fig. 4:**
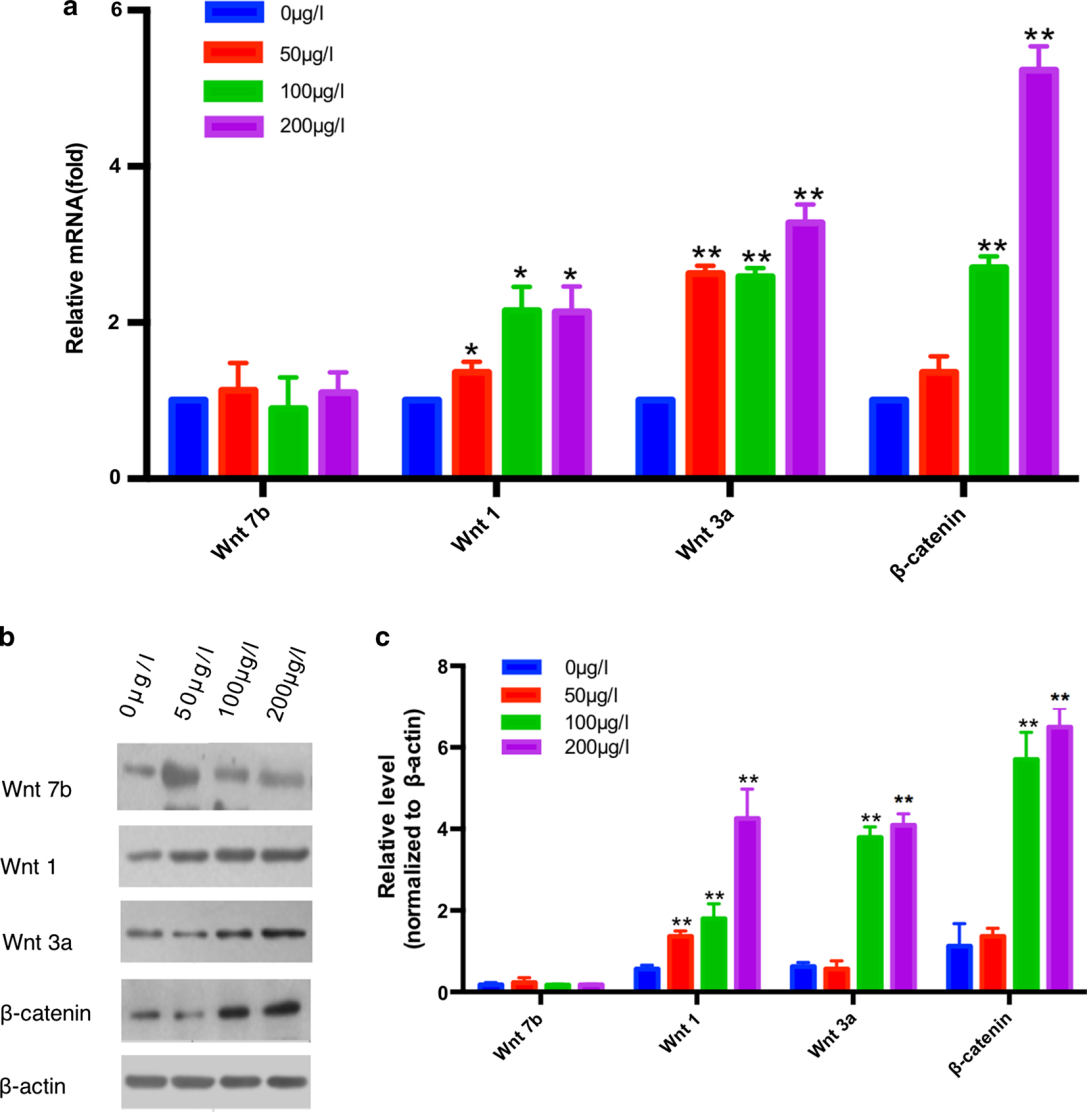
Periostin suppressed the osteogenic differentiation of suture-derived cells via inactivation of Wnt/β-catenin signaling. **a** The mRNA expression of Wnt-7b, Wnt-1, Wnt-3a, and β-catenin of suture-derived cells after treatment with different concentrations of recombinant mouse periostin. **b** Western blotting analysis of Wnt/β-catenin signaling markers. **c** Quantification of Western blotting results. *P < 0.05, **P < 0.01 compared to 0 μg/L group

We next studied the molecular mechanism of how recombinant mouse periostin regulates osteogenic differentiation functions, and we found the stimulator of Wnt canonical signaling: specifically, with regard to, Wnt-3a, Wnt-1, and β-catenin, the molecular node of the Wnt/β-catenin signaling was upregulated. The expression of Wnt-7b was not affected As shown in Fig. 4a, the expression levels of Wnt-1 and Wnt-3a in the 50 μg/l group, 100 μg/l group, and 200 μg/l group were all higher than those in the 0 μg/l group (p < 0.05, p < 0.01) and the expression levels of β-catenin in the 100 μg/l group and 200 μg/l group were significantly increased (p < 0.01). These results were also proven by Western blotting (Fig. 4b, c).

### Periostin ameliorates coronal synostosis in Twist1^+/−^ mice in vivo

**Fig. 5:**
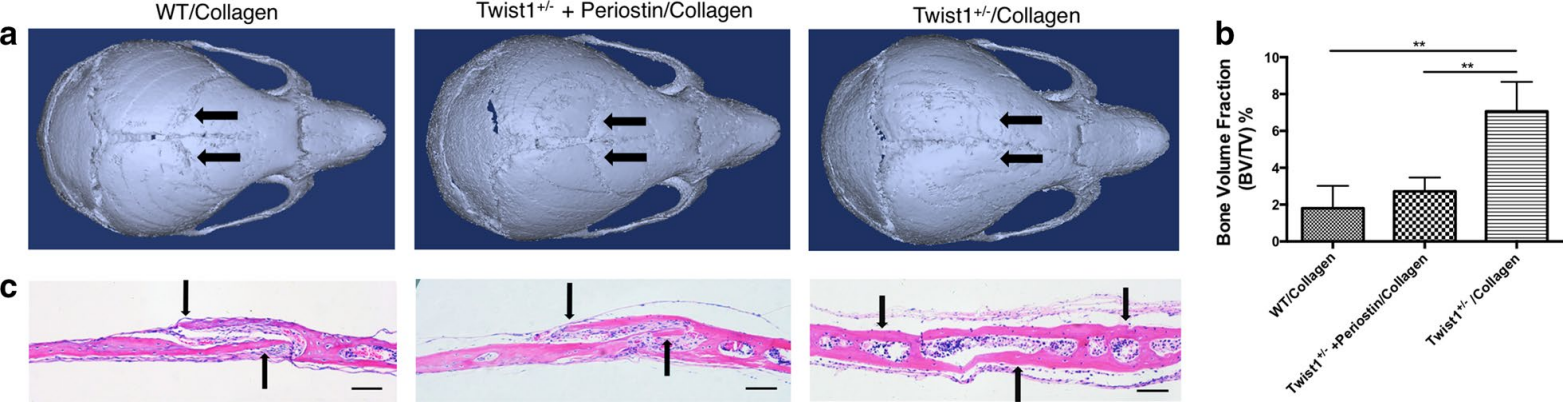
Periostin ameliorates coronal synostosis in Twist1^+/−^ mice in vivo. **a** Micro-CT shows that recombinant mouse periostin can suppress the coronal suture synostosis of Twist1^+/−^ mice (arrows, coronal suture). **b** Coronal sutures bone volume fraction analysis. After treatment with recombinant mouse periostin, the bone volume fraction of coronal suture decreased significantly (p < 0.01). **c** Hematoxylin and eosin staining to observe the osteogenic fronts of coronal sutures; the osteogenic fronts of the Twist1^+/−^ mice remain detached after treatment for 3 weeks (arrows, osteogenic fronts). Original magnifications: ×50. Bar: 50 μm. *p < 0.05, **p < 0.01

Micro-CT imaging revealed patent coronal sutures in wild-type mice and periostin-treated Twist1^+/−^ mice; however, the results showed coronal synostosis in mutant mice (Fig. 5a). Haemotoxylin and eosin staining showed abnormal development of the Twist1^+/−^ mice coronal sutures with presynostosis and osteoid deposition between the osteogenic fronts (Fig. 5c, arrows, osteogenic fronts); however, the bony edges of the coronal sutures in both wild-type mice and periostin-treated Twist1^+/−^ mice remained detached (Fig. 5c). We also tested the bone volume fraction of coronal sutures, and the results showed that in comparison with control Twist1^+/−^ mice (7.13 ± 1.25%), the bone volume fraction of periostin-treated Twist1^+/−^ mice (2.41 ± 0.68%) decreased significantly (p < 0.01) to a level that was a little higher than the bone density of wild-type mice (1.98 ± 0.97%), and that there were no significant differences of bone density the between wild-type mice and periostin-treated Twist1^+/−^ mice (Fig. 5b).

## Discussion

SCS is a type of ACS syndrome, which is characterized by the premature fusion of the coronal sutures. The majority of the mutations that have been described to date result in a loss of function, leading to functional haploinsufficiency of TWIST1. To date, more than 100 distinct mutations in the TWIST1 gene have been found to cause SCS, including nucleotide substitutions (missense and nonsense), deletions, insertions, duplications, and complex re-arrangements [15]. TWIST1 is a transcription factor that belongs to the bHLH family. Structurally, bHLH proteins are characterized by the presence of a conserved domain containing a stretch of basic amino acids adjacent to two amphipathic α-helices separated by an inter-helical loop [16]. In addition, TWIST1 is primarily expressed in adult stem cells located in mesoderm-derived mesenchymal tissues, such as bone [17], muscle [18], adipose tissue [19], and bone marrow [20]. Therefore, TWIST1 is especially important to the morphology and behavior of the head mesenchyme cells that support morphogenesis of the cranial neural tube.

As the primary treatment for SCS remains surgical in nature, a greater understanding of these and other pathways could lead to the development of innovative approaches for applying alternative medical therapies to the treatment of craniosynostosis, in particular by maintaining suture patency. The identification of signaling pathways pathologically activated in the cranial suture raises the possibility of the use of adjuvant medical therapies in the future.

In order to study related molecules in craniosynostosis, we identified the fact that there is a low expression of periostin in the fusion sutures of craniosynostosis patients and Twist1^+/−^ mice. Periostin is expressed in bone, skin, tendons, neoplastic tissue, cardiovascular and fibrotic diseases. It is also a significant marker of osteoblast precursors in the periosteum and its expression is decreased in the process of osteoblast differentiation [21]. Therefore, we speculated that periostin may influence the biological behavior of suture-derived cells and ameliorate craniosynostosis in Twist1^+/−^ mice. Twist1^+/−^mice serve as an important mouse model for SCS. Our mouse model showed that the coronal suture completely closed at 10 days after birth, which is similar in SCS patients (Additional file 1: Figure S1). Our results agree with those of other studies [22–24].

We observed that recombinant mouse periostin inhibited the proliferation of suture-derived cells. The effects showed progress in a concentration- and time-dependent manner and were most significant when the concentration was 200 μg/l—at this point, the migration ability of suture-derived cells was also inhibited. Additionally, the presence of periostin seems to resist the progress-enhanced mineralization, with osteoblast markers decreased. The expressions of ALP, BSP, COL-I, and OCN were decreased significantly. The in vivo results showed our periostin-treated mice presented incomplete coronal cranial suture closure and displayed patency of the posterior frontal suture and sagittal suture. We interpret these findings to indicate that periostin was involved in tissue growth or mineralization, or both, and that it plays an important role in coronal suture growth and coronal cranial suture closure. Therefore, we firmly believe that the therapeutic attributes of periostin would improve SCS.

It has been demonstrated that the loss-of-function mutations of TWIST1 have led to increased mineralization in osteoblasts derived from patients with SCS at days 21 and 28 in culture [25]. Here, we observed that the suture-derived cells’ proliferation and osteogenic differentiation were suppressed by perisostin. Moreover, reporter analyses indicated that the periostin promoter activities were enhanced by the overexpression of TWIST1 and that such can bind to the periostin promoter in undifferentiated preosteoblasts and upregulate periostin expression [26]. Therefore, we speculated the periostin may rescue the loss of TWIST1 in suture-derived cells and normalize elevated proliferation and mineralization. It has also been reported that the effects of periostin on osteoblast development differ depending on the stage of the osteoblast differentiation target [27]. Thapa et al. found that βig-h3 protein, a molecule-bearing fasciclin domains that is highly homologous to periostin, inhibits bone nodule formation of osteoblasts in vitro [28]. These studies support our results. Previous literature showed that the migration of calvarial osteoblasts plays a crucial role in the patterned growth of calvarial bones and that abnormal migration of these cells can lead to the development of craniosynostosis [29]. We found that there was a significant decrease in cell migration with periostin treatment. It has been clarified that the osteogenic rudiments and the prospective coronal suture expand apically by cell migration [30]. Periostin also suppresses cell migration in human bladder cancer cells via a TAB 1(TGF-beta activated kinase)/TAK1 (TGF-beta activated kinase 1) signaling pathway [31], providing a possible explanation for its potent role in controlling the migration of suture-derived cells.

However, the expression of RUNX-2 was not affected after treated with periostin in Twist1^+/−^ cells; as a matter of fact, TWIST1 can bind to the periostin promoter in undifferentiated preosteoblasts and upregulate periostin expression [27]. Many examples in the literature showed that TWIST1 also suppresses the activity of RUNX-2 and thereby regulates bone formation [32], which may suggest that TWIST1 can act independently to stimulate osteogenesis or periostin may change the activity of RUNX-2 but not its expression to inhibit osteogenic differentiation in sutured-derived cells. Many reports have also demonstrated that periostin is expressed early in osteoblasts and is involved in the differentiation process, possibly through integrin binding [10, 33, 34]; however, the mechanisms involved in periostin/integrin binding in bone have not been analyzed yet in detail. In this study, we found that periostin suppressed the osteogenic differentiation of suture-derived cells, which is a new discovery that has not previously been reported.

To fully elucidate the underlying the mechanism about how recombinant mouse periostin inhibits the osteogenic differentiation of sutured mesenchymal cells, we examined the role of Wnt/β-catenin signaling. Wnt-7b, Wnt-1 and Wnt-3a are wnt ligands of the wnt signaling pathway. Additionally, Wnt-7b, Wnt-3a and Wnt-1 are both stimulators of Wnt canonical signaling that act by directly binding to Wnt ligands or Frizzled receptors [35]. β-catenin is the most critical factor of the Wnt signaling pathway: in the cytoplasm, when the Wnt signaling pathway is not activated, β-catenin will be phosphorylated to initiate the ubiquitin system and degraded by the proteasome pathway. In the nucleus, β-catenin binds to a nuclear transcription factor (TCF/lEF) to initiate the transcription of target genes, such as c-myc and cyclinD [36]. The pronounced expression of Wnt-3a, Wnt-1, and β-catenin suggests an upregulation of Wnt signaling while the expression of Wnt-7b was not affected. Several studies have found that there are significant relationships between periostin and the Wnt/β-catenin pathway. Lv et al. demonstrated that increased periostin expression can subsequently inhibit the expression of sclerostin and activate Wnt/β-catenin signaling pathways in bone tissue [37]. Additionally, periostin was also shown to activate Wnt/β-catenin signaling in primary osteoblastic cell cultures and in TOPGAL reporter mice [38]. This is consistent with our results that the increased expression of Wnt-3a, Wnt-1, and β-catenin result from treatment with periostin. The role of Wnt signaling in craniosynostosis has been previously reported. Behr [39] discovered that, during the first month of life, the murine posterior-frontal suture of the cranial vault closes through endochondral ossification, while other sutures remain patent. These processes are tightly regulated by canonical Wnt signaling. Low levels of active canonical Wnt signaling enable endochondral ossification and therefore posterior-frontal-suture closure, whereas constitutive activation of canonical Wnt causes posterior-frontal-suture patency [40]. Zhang et al. [41] showed that the Wnt/β-catenin pathway is inhibited in both Apert syndrome mouse mutant bone marrow stromal cells and osteoblasts, and differentiation defects of these cells can be ameliorated by Wnt-3a treatment. All of the studies suggest that the deregulation of the Wnt/β-catenin pathway may underlie craniosynostosis. Our use of periostin on suture-derived cells may inhibit the osteogenic differentiation via activating the Wnt/β-catenin pathway.

## Conclusions

In summary, we have discovered that recombinant mouse periostin inhibited osteogenesis in coronal suture-derived cells of a Twist1^+/−^ mouse model. This in vitro phenomenon is accompanied by suppressive proliferation and cell migration, and in vivo recombinant mouse periostin treatment applied in Twist1^+/−^ mice also appeared to be patent in coronal sutures. These in vitro and in vivo data have implications for determining the mechanisms of periostin in coronal craniosynostosis of patients with SCS.

## Additional file

**Additional file 1: Figure S1.** The coronal suture of Twist1^+/−^ mice shows fusing at P1, but with fusion occurring 10 days after birth. The wild-type mouse remains patent during this period.

## Abbreviations

TWIST1: twist family basic helix-loop-helix transcription factor 1
ALP: alkaline phosphatase
BSP: bone sialoprotein
OCN: osteocalcin
COL-1: collagen type I
RUNX-2: runt-related transcription factor 2
ACS III: acrocephalosyndactyly III
SCS: Saethre–Chotzen syndrome
CCK-8: Cell Counting Kit-8
RT-qPCR: real-time quantitative polymerase chain reaction
DAPI: 4′,6-diamidino-2-phenylindole
PBS: phosphate buffered saline
CT: computed tomography
FGFR: fibroblast growth factor receptor
bHLH: basic helix-loop-helix
