# Supplementary figures and images for: Recombinant mouse periostin ameliorates coronal sutures fusion in Twist1+/− mice
Source: J Transl Med. 2018 Apr 17;16:103. doi: 10.1186/s12967-018-1454-2 (PMC5905175; doi:10.1186/s12967-018-1454-2)

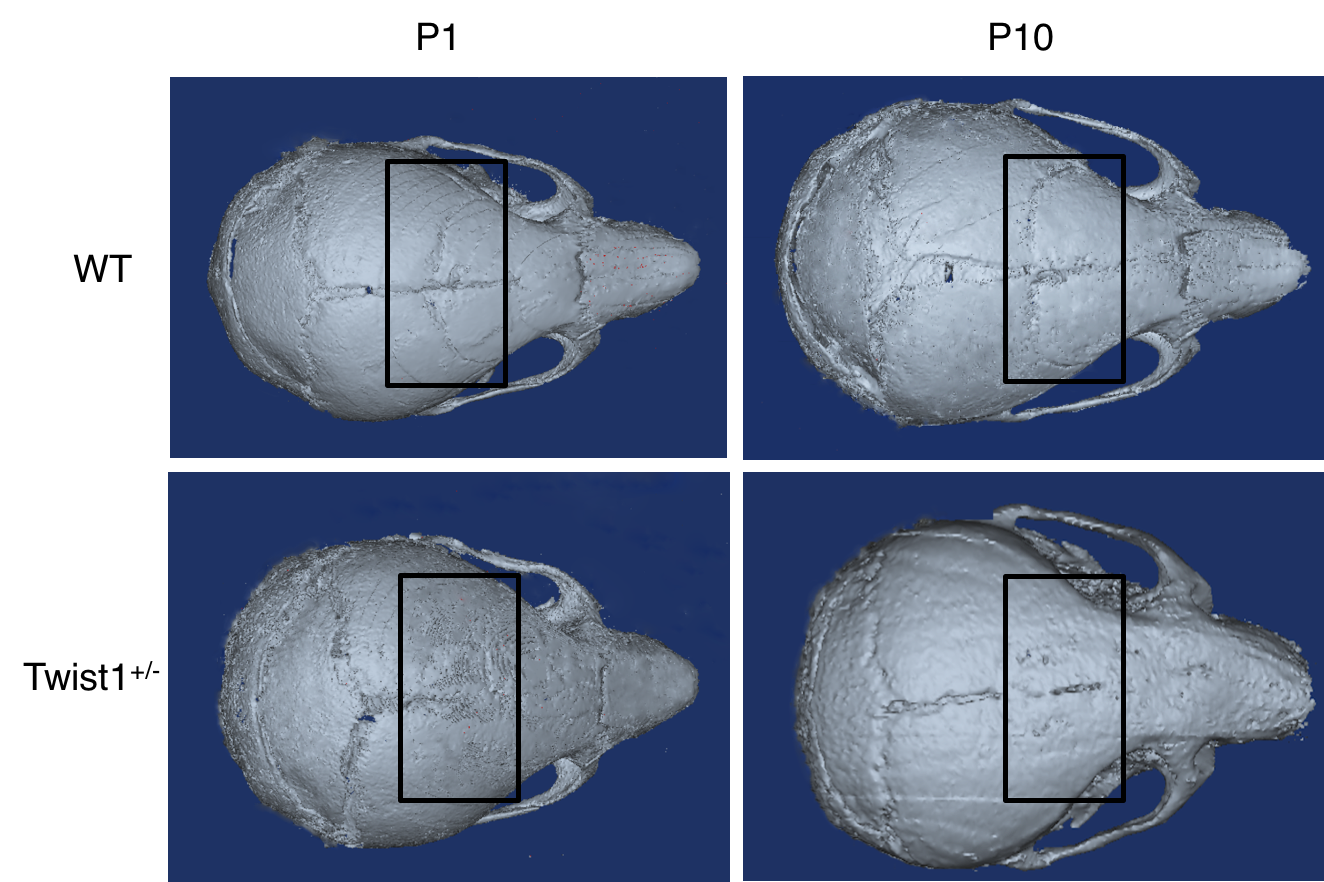

Supplement: Supplementary file 1 — Additional file 1: Figure S1. The coronal suture of Twist1+/− mice shows fusing at P1, but with fusion occurring 10 days after birth. The wild-type mouse remains patent during this period. [file 12967_2018_1454_MOESM1_ESM.tif]
